# *De novo* transcriptomic analysis of Doum Palm (*Hyphaene compress*a) revealed an insight into its potential drought tolerance

**DOI:** 10.1371/journal.pone.0292543

**Published:** 2024-03-12

**Authors:** Allen Johnny Borlay, Cecilia Mbithe Mweu, Steven Ger Nyanjom, Kevin Mbogo Omolo, Labode Hospice Stevenson Naitchede

**Affiliations:** 1 Department of Biological Sciences, University of Liberia, Monrovia, Liberia; 2 Department of Molecular Biology and Biotechnology, Pan African University Institute for Basic Sciences, Technology and Innovation, Nairobi, Kenya; 3 Institute for Biotechnology Research, Jomo Kenyatta University of Agriculture and Technology, Nairobi, Kenya; 4 Department of Biochemistry, Jomo Kenyatta University of Agriculture and Technology, Nairobi, Kenya; University of Agricultural Sciences, INDIA

## Abstract

**Background:**

Doum palms (*Hyphaene compressa*) perform a crucial starring role in the lives of Kenya’s arid and semi-arid people for empowerment and sustenance. Despite the crop’s potential for economic gain, there is a lack of genetic resources and detailed information about its domestication at the molecular level. Given the doum palm’s vast potential as a widely distributed plant in semi-arid and arid climates and a source of many applications, coupled with the current changing climate scenario, it is essential to understand the molecular processes that provide drought resistance to this plant.

**Results:**

Assembly of the first transcriptome of doum palms subjected to water stress generated about 39.97 Gb of RNA-Seq data. The assembled transcriptome revealed 193,167 unigenes with an average length of 1655 bp, with 128,708 (66.63%) successfully annotated in seven public databases. Unigenes exhibited significant differentially expressed genes (DEGs) in well-watered and stressed-treated plants, with 45071 and 42457 accounting for up-regulated and down-regulated DEGs, respectively. GO term, KEGG, and KOG analysis showed that DEGs were functionally enriched cellular processes, metabolic processes, cellular and catalytic activity, metabolism, genetic information processing, signal transduction mechanisms, and posttranslational modification pathways. Transcription factors (TF), such as the MYB, WRKY, NAC family, FAR1, B3, bHLH, and bZIP, were the prominent TF families identified as doum palm DEGs encoding drought stress tolerance.

**Conclusions:**

This study provides a complete understanding of DEGs involved in drought stress at the transcriptome level in doum palms. This research is, therefore, the foundation for the characterization of potential genes, leading to a clear understanding of its drought stress responses and providing resources for improved genetic modification.

## 1 Introduction

Stress from drought is among the most severe forms of environmental limitation, as it has a chilling effect on plant development and productivity. Worldwide, agriculture is becoming increasingly challenging to grow food in areas experiencing more extended periods of water stress due to climate change. Drought may induce organic process disproportion in plant cells and alter plant leaves’ optical energy assimilation, damaging plants’ photosynthetic organs [1]. Plants frequently encounter uncomplimentary environmental circumstances throughout their lifetime due to their sessile nature, and these circumstances, which include drought, restrict plant development and output [2]. Plant researchers and breeders are keenly interested in the plant’s entire cellular workings that govern its reaction to drought stress to sustain and improve crop yields. Understanding the mechanisms that boost plants during drought is crucial for designing new drought-resistant strategies and maintaining global food security [3].

Plants have evolved various self-defense and defensive systems to deal with drought stress throughout their evolutionary process [4]. As indicated by [5], many genes are expressed in plants in retort to water deficit. Genes for protein structural maintenance, cell-water binding capacity, heat shock proteins, proteins prevalent in late embryogenesis, and genes that keep membrane proteins stable are some of the genes that help plants retort to drought. Plants are protected from the harmful effects of water shortages by proteins that change how genes are expressed and control metabolic processes. The synthesis of these proteins is a defense mechanism against the first effects of water deficit. Furthermore, transcription factors (TFs) normalize the workings of numerous genes to thwart these negative consequences [6]. Determining how crops can tolerate water deficits and creating new methods of plant cultivation to resist climate change would require understanding the genes translated and expressed in reaction to water stress.

Doum palms (*Hyphaene compressa*) perform a crucial starring role in the lives of Kenya’s arid and semi-arid people (Nomadic pastoralists) for economic empowerment and sustenance [7]. During the lingering summertime, when there is widespread food insecurity across Africa, especially in Djibouti, Sudan, Kenya, Nigeria, and Namibia, the fruits of Hyphaene are considered an essential source of subsistence [8, 9]. Doum palm fruit aqueous extract possesses anti-inflammatory, antibacterial, and anticancer phytoconstituents [10]. In Kenya, the leaves of Doum Palm are used for making thatch, baskets, mats (tablemats, floor mats, sleeping mats), brooms, carpets, ropes, caps, seats, or door shutters.

Due to their unique adaptation to water scarcity and dry climates, doum palms can be found in Kenya’s arid and semi-arid regions, particularly in the north river courses, lakes, and coasts [11]. *Hyphaene* species can be found in various habitats, including sandy lowlands, secondary woods open to the sky, and coastal and inland savannahs [8]. Coastal savannas in East Africa, particularly in Kenya and Tanzania, are dominated by their distinctive shapes.

Recent advances in molecular biology techniques have helped find plants’ possible environmental stress response mechanisms. RNA Sequencing (RNA-Seq), a method for profiling expressed transcripts, is used to determine essential genes/factors related to abiotic/biotic stresses or diseases in plants and other species [12]. There are apparent advantages to using RNA-Seq, including the ability to carry out large-scale functional gene assignment, qualitative and quantitative gene expression analysis, and improved understanding and precision in characterizing eukaryotic transcriptomes, particularly those from non-model species [13]. RNA-Seq has changed plant transcriptomics research since it is efficient, reliable, and effective. Many plant researchers and molecular breeder have successfully used RNA-Seq to classify abiotic condition retort mechanisms. This powerful technology is widely used to sequence transcriptomes, allowing for the generation of functional genomic data and the discovery of DEGs across cultivars, organs, and treatment conditions [14–16]. RNA-seq data, such as annotated genes, DEGs, and molecular markers, may offer insight into discovering novel genes [17]. Here, high automated sequencing technique was utilized to sequence the transcriptomes of drought-stressed leaf samples from doum palms to obtain functional genomic data that may shed light on the molecular processes underpinning drought responses in doum palms.

The transcriptomes of doum palms responding to drought stress have been systematically characterized in this work. These doum palm transcriptome data can potentially be exploited in crop enhancement strategies in the molecular breeding of doum palms for abiotic stress tolerance by identifying genes and markers involved in water deficit tolerance.

## 2 Materials and methods

### 2.1 Doum palm samples and stress imposition for *de novo* assay

Doum palm seeds were collected from Tharaka Nithi, Kenya, in May of 2021 and brought to the Institute for Biotechnology Research (IBR) greenhouse at the Jomo Kenyatta University of Agriculture and Technology (JKUAT) for further use. Doum palms are wild plants hence no permission is needed for seed collections as per the Kenya laws. Additionally, appropriate greenhouse guidelines and procedures were straightly followed as provided by the JKUAT charter. Before sowing, the seeds were unshelled using a knife to break seed dormancy (physical application by using a knife and applying force to remove the outer covering of the doum palms seeds) and surface sterilized by successive washings using 3% redomil and distilled water. Seedlings were soaked in distilled water for 72 hours to induce rooting until germination. After seven weeks, the germinated seedlings were transplanted and grown in pots with regular watering for ten months before being subjected to drought stress for 65 days with fully irrigated samples as a control. The control plants received constant watering (1.5 liters) every two days, while the drought-stressed plants received no water for 65 days with a greenhouse temperature of 26°C.

### 2.2 Total RNA isolation and cDNA synthesis

RNA was extracted using a modified ISOLATE II Plant Kit (Meridian Bioscience, London, UK).

Trizol (600 μL) was used to lyse up to 100 mg of tissue at a time. 250 μL of membrane desalting buffer (MEM) was use to desalt the silica membrane. RNA extracts were treated with DNAse enzyme for 15 minutes at 20°C to eliminate contaminating DNA. RNase-free water (50 μL) was used to elute the RNA. Jenway Genova Nanodrop Spectrophotometer (Bibby Scientific Ltd Beacon, United Kingdom) and the MyGel Mini compact electrophoresis system (Benchmark Scientific Inc, Sayreville, NJ 08872 USA) were used to examine the isolated RNA for concentration, purity, and quality. Using a TetroTM cDNA Synthesis Kit, the isolated RNA was converted into cDNA by reverse transcription (Meridian Bioscience, London, UK).

### 2.3 Library construction, sequencing, reads filtering, and *De novo* assembly

The Beijing Genomic Institution performed (BGI) the library construction and sequencing on a BGISEQ-500 platform. *De novo* assembly with clean readings was accomplished using the Trinity package with Kmer = 25 as the standard parameter [18]. Using Tgicl, the assembled transcripts were grouped hierarchically [19]. Raw sequence reads were subjected to quality filtering using FasqQC (version 0.11.5). Raw reads are a characteristic of low-quality reads, adapter contamination, and a large concentration of unknown base (N) reads, which should be filtered out before further analysis. To get more accurate results and clean readings, we discarded short reads containing adapters, reads with values below 5, and reads with unknown bases (N) over 10% of the total bases. Clean data were calculated simultaneously, including GC content, Q20, Q30, and sequence repetition levels. The *de novo* transcriptome short reads and unigene refer to the sequences generated by Trinity’s method.

Contigs were formed from the overlap regions of the short reads, and for the final set of Unigenes, gene family clustering was performed via Tgicl. Following hierarchical clustering, each cluster’s lengthiest sequence (unigene) was utilized for further data on length circulation, gene identification, and DEG detection. The unigenes were then categorized into gene families, following which clusters and singletons were discovered as two distinct categories.

### 2.4 Functional annotation, open reading frames (ORFs), and Pfam domain detection

For gene annotation, the BLAST (E-value < 1.0 × 10^−5^) programs [20] and Diamond [21] against NT (Nucleotide database), NR (Protein database), KOG, KEGG, and SwissProt databases were utilized. The [22] method was used to annotate the InterPro database, and Blast2GO [23] was used to annotate the NR and GO terms. Sequence-identical proteins to the supplied unigenes were retrieved with annotations on their putative functions. The degree of association between biological repeats was calculated using the Pearson’s correlation analysis on fragments per kilobase per million fragments (FPKM) values from reads. The possible coding region was subsequently determined using the TransDecoder application. Predicted coding sequences (CDSs) were obtained from Pfam homology sequences by blasting the most extended open reading frame (ORF) against the SwissProt and Hmmscan Coding DNA sequence databases. Furthermore, query sequences derived from unigenes were utilized to search the Pfam (Protein family) database.

### 2.5 Unigene Gene Ontology (GO) Classification and Enrichment Analysis for DEGs

The GO annotation of the unigenes was obtained using the NR with the application of the Blast2GO program. GO functional evaluation was carryout for the entire transcripts with the aid of the WEGO program to get a global view of the distribution of genes’ roles in doum palms [24]. A gene list and gene counts for each of the DEGs annotated with GO terms were generated by first mapping all of the DEGs in the GO catalog. The hypergeometric test was utilized to discover highly improved GO terms among the DEGs using the formula below to calculate the p-value:

$$\text{P}=1-\sum_{i=0}^{m-1}\frac{\left(\begin{array}{c} M\\ i \end{array}\right)\left(\begin{array}{c} N-M\\ n-i \end{array}\right)}{\left(\begin{array}{c} N\\ n \end{array}\right)}$$

### 2.6 Kyoto encyclopedia of genes and genomes (KEGG) pathway evaluation for DEGs

After considering the complete transcriptome, we find that enrichment analysis significantly enhanced specific pathways regarding DEGs. We utilized the p-value technique that mirrors the one applied in the GO term analysis. An analog of the p-value known as Q represents the FDR (false discovery rate) equivalent of a value of 0.01. Multiple test adjustments allow us to identify highly enriched DEG pathways with Q-values of ≤0.05.

### 2.7 Transcription factor (TF) analysis of DEGs and simple sequence repeats (SSR) detection

We used the getorf program [25] to determine the open reading frame (ORF) of each Unigene, and then we used hmmsearch [26] to align the ORF to TF realms from the PlntfDB database, thereby identifying each TF following the PlantfDB standards. SSRs in the unigenes of doum palm were detected using the MIcroSAtellite program with the default setting.

### 2.8 Gene expression quantification and DEGs screening

To get the number of reads count, we used the Bowtie aligner and the expectation-maximization method (RSEM) from the Trinity protocol to run the Perl script align and estimate its richness. Through assembly and clustering, FPKM scores representing relative quantification levels of individual unigene transcripts were determined using the normalization procedure [27] approach. We used DESeq2, an R package developed by [28], to analyze the DEGs. DEGs fold-change (FC) values were calculated based on the following cutoff criteria: P<0.05 and log |FC|≥1:5. Significant positive or negative changes in logarithmic values indicated the DEGs’ up-regulated or down-regulated status.

### 2.9 Quantitative RT-PCR authentication of sequencing results

We utilized a CFX Connect qPCR detection instrument to confirm the sequencing findings for gene expression profiling. The elongation factor 1-alpha gene (accession number: AB061263) served as a reference gene, while four drought tolerance-related genes were randomly chosen (Table 1) for validating the RNA-Seq results. The NCBI Primer-BLAST program, a resource for generating gene-specific primers, was utilized to brand primers for evaluating relative gene expression. After that, validation analysis was performed in 96 well-plates (Applied Biosystems, Foster, California) with SYBR Green master mix (Luna^®^ Universal qPCR Master Mix, NEB) in qTower (Applied Biosystems) at 95°C for 60 seconds, proceeded by 40 cycles of 95°C for 15 seconds, and 58°C for 30 seconds. The gene expression level was performed by the comparative 2^−ΔΔ^ CT methods [29] using all samples in triplicate for normalization.

**Table 1:** Gene used for RNA-Seq validation.

Primer ID	Gene accession	Forward sequence	Reverse sequence	Size
**Efl α**	AB061263	ATTGGAAACGGATATGCTCCA	TCCTTACCTGAACGCCTGTCA	188
**CL5334.Contig3**	XP_008794197.1	GATCTTACCAAGCGCCAGCA	CCTGGGAATGCACAAGCAAG	140
**CL5518.Contig6**	XP_010920097.1	CATCCTCGTCTACTGCACCG	ATGATCCCCTTGACGATGCC	165
**CL1905.Contig16**	XP_010926195.1	GACCCCTCGTCTGAAGGTTG	CTTTGGACCTGCTTGCGAAC	254
**CL8906.Contig36**	XP_010916565.1	CCGGACTCGTTTGGGAAACT	TCCCCTCCTTTCCAGTCAGA	209

#### 2.9.1 Sample size, *De novo*, and qPCR statistical analysis

The experiment used a completely randomized design (CRD) setup with three biological and technical replicates for each treatment. The setup had two treatments: drought/stress and control/well-watered. There were 18 individual data points (3 biological replicates x 3 technical replicates x 2 treatments) used for quality control and assessing variability representing the total samples or dataset (a representative of the treatments and replicates). We used SAS version 9.2 and GraphPad Prism 8 for the statistical analysis. A one-way analysis of variance (ANOVA) with Tukey’s post hoc test was employed to identify any significant differences between groups and treatments. All experimental findings were presented as means with standard deviations (SDs) with a significance threshold for statistical analysis set at 0.05.

## 3 Results

### 3.1 Validation of extracted total RNA and cDNA synthesis

This work validated extracted total RNA using qualitative and quantitative nucleic acid detection techniques. High brightness and clarity in the 28S and 18S stripes and the absence of a towing degradation characteristic indicate intact RNA. The Nanodrop spectrophotometer results from the ISOLATE II Plant extraction Kit showed total RNA isolated from doum palm leaves, A260/A230 between 1.92 and 2.13, and concentrations ranging from 579.61 to 1601.4 ng/μL

### 3.2 Sequence data summary and *de novo* assembly

The raw reads from the sequenced doum palm leaves were uploaded to the Genebank portal at NCBI with the accession number PRJNA861903. The raw data generated was approximately 39.97 Gigabytes (Gb). The quality control (QC) results based on an average sequencing error rate of 0.01% showed that the sequence data had a median of 96.41%, 88.76%, and 42.83% for Q20, Q30, and GC content, respectively (Table 2).

**Table 2:** Clean reads quality data.

Sample	C	D	J	K	M	N	Average
Total Raw Reads (M)	50.83	50.83	50.83	50.83	52.59	50.83	51.12
Total Clean Reads (M)	44.11	44.18	44.06	44.34	45.27	44.47	44.41
Total Clean Bases (Gb)	6.62	6.63	6.61	6.65	6.79	6.67	6.66
Clean Reads Q20 (%)	96.47	96.52	96.32	96.25	96.46	96.41	96.41
Clean ReadsQ30 (%)	88.94	89.08	88.52	88.36	88.9	88.73	88.76
Clean Reads Ratio (%)	86.77	86.91	86.68	87.23	86.08	87.48	86.86
GC (%)	42.91	42.92	42.94	42.22	42.96	43	42.83

D, J, and M are control samples; C, K, and N are stress samples

*De novo* assembly with Trinity yielded 193,167 unigenes with an average length of 1655 bp and an N50 of 3165 bp (Table 3 and S2 Fig). The results of the NR database function annotation allowed us to determine the species proportion on Unigene annotation and created a distribution map, which showed that 51.8% of the annotations were for *Phoenix dactylifera*, 39.19% were for *Elaeis guineensis*, and 1.08% were for *Ananas comosus*, with the remaining 7.85% being for other species (S1 Fig).

**Table 3:** *De novo* assembly of clean reads.

Sample	Total Number	Total Length	Mean Length	N50	N70	N90
C	241,065	202,188,614	838	1,571	829	306
D	262,476	215,137,283	819	1,590	803	293
J	249,710	206,523,523	827	1,594	816	297
K	288,674	244,491,248	846	1,647	847	302
M	274,264	218,897,862	798	1,575	775	281
N	244,877	197,684,025	807	1,577	792	287
**All Unigene**	**193,167**	**319,715,115**	**1,655**	**3,167**	**1,997**	**759**

N50: Is a weighted median statistic that 50% of the total length is contained in Unigenes that are equal to or larger than this value

N90: The length for which the collection of all contigs of that length or longer contains at least 90% of the sum of the lengths of all contigs

### 3.3 Doum palm annotation matrics with public databases

We aligned our sequence data in various databases, including the protein sequence (NR), Nucleotide Sequence Database (NT), KEGG, Swiss-Prot, Interpro GO, and eukaryotic orthologous groups (KOG) for homologs search of the assembled unigenes (Table 5 and S3 Fig). There were 128,708 unigenes effectively annotated with at least one database, including 115,388 (73%), 122,767 (63.55%), 75,426 (39.05%), 91,401 (47.32%), 89,403 (46.28%), 92,118 (47.69%) and 23,074 (11.95%) that were significantly matched with the NR, NT, Swissprot, KEGG, KOG, Interpro and GO databases (Table 4 and S3 Fig).

**Table 4:** Function annotation summary.

Values	Total	NR	NT	Swissprot	KEGG	KOG	Interpro	GO	Intersection	Overall
Number	193,167	115,388	122,767	75,426	91,401	89,403	92,118	23,074	12,760	128,708
**Percent**	100%	59.73%	63.55%	39.05%	47.32%	46.28%	47.69%	11.95%	6.61%	**66.63%**

**Intersection:** The total amount of Unigenes annotated

**Overall:** Quantity of annotated Unigenes

**Table 5:** Doum palm predicted CDS quality.

Total #	Total length	N50	N90	Max Length	Min Length	Sequence (GC%)
94741	108,239,283	1,476	567	15,321	279	46.41%

Total number: the number of CDS

#### 3.3.1 Functional KEGG annotation in doum palm

**Fig 1:**
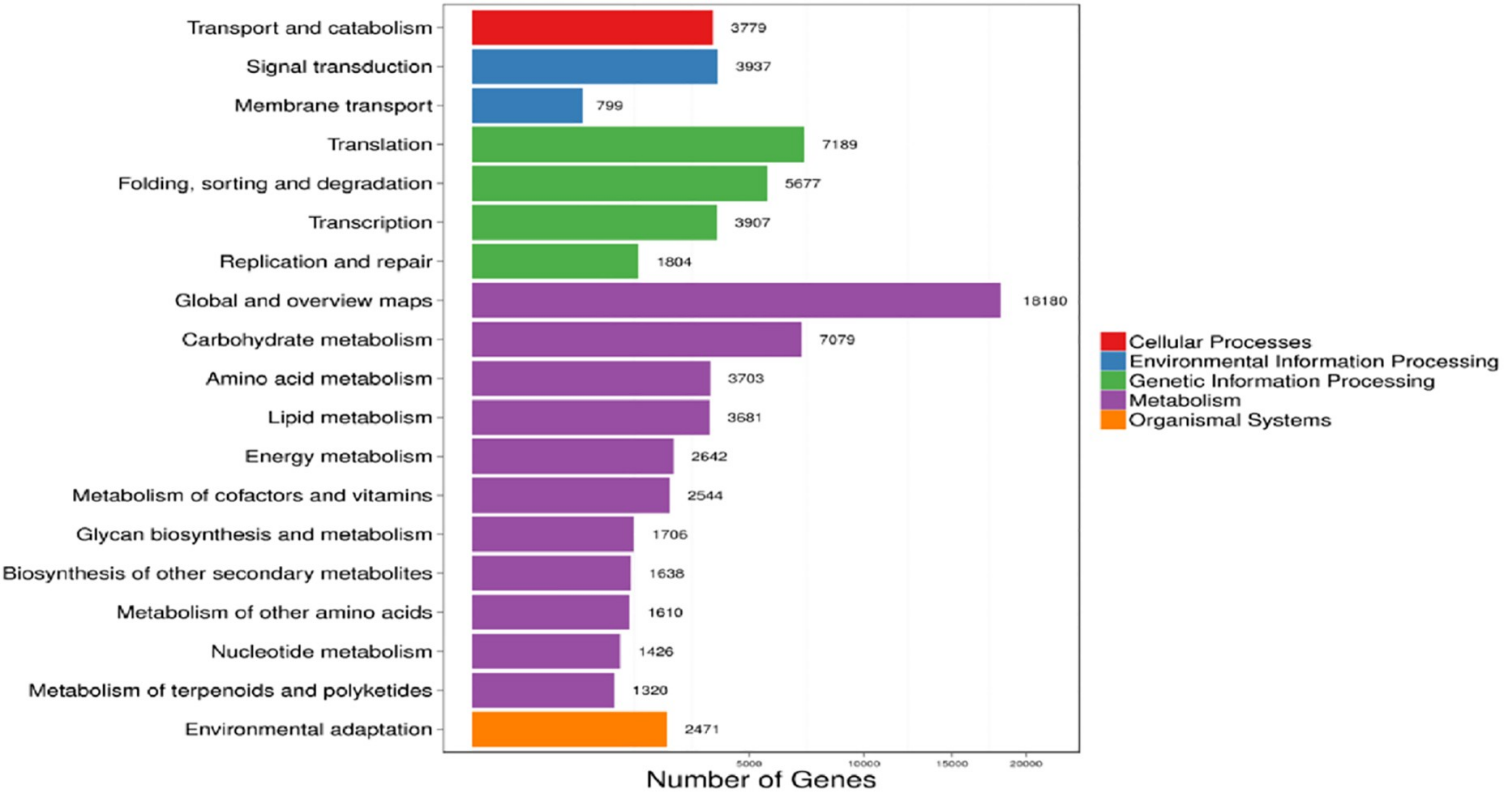
KEGG annotation functional distribution. Horizontal and vertical bars indicate the sum of unigenes and KEGG functional grouping, respectively. Different color represents five of the seven categories of the KEGG metabolic pathway.

The KEGG documented five of the seven critical metabolic pathway components (KEGG) in the doum palm transcriptome analysis. Functional categories of the Unigenes studied here included cellular activities, environmental information processing, genetic information processing, metabolism, and organismal systems (Fig 1). Analysis of the doum palm transcriptome via the KEGG showed that "metabolism" and "genetic information processing" accounted for the most significant categories. Our initial impression is that the sequencing and assembly methods employed here are of the highest quality. In the metabolism category, "global and overview maps" accounted for 18180 genes, while carbohydrate metabolism was followed by coding for 7079 genes. The genetic information processing category coded 7189 and 5677 genes representing translation and "folding, sorting and degradation," respectively (Fig 1).

#### 3.3.2 Eukaryotic Orthologous Groups (KOG) functional classification in doum palm

**Fig 2:**
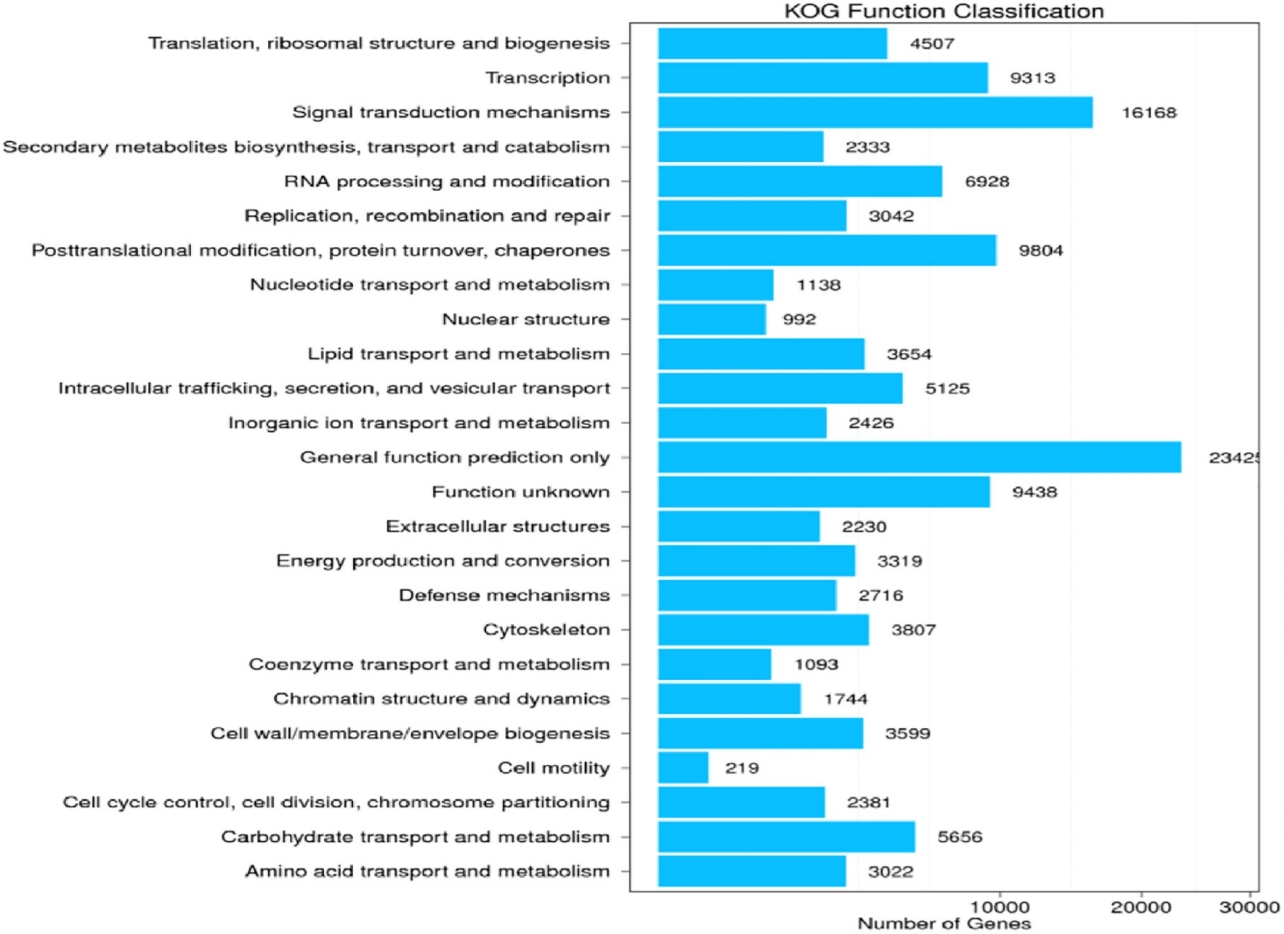
KOG functional classification. The abscissa and vertical coordinate depict the amount of Unigenes and KOG functional categories, respectively.

Fig 2 shows the distribution of annotated Unigenes in the KOG database according to the 25 functional categories. The functional distribution result shows that "general function prediction (23425 genes)", "signal transduction mechanisms (16168 genes)", "posttranslational modification, protein turnover, chaperones (9804 genes)," "unknown function (9438 genes)", and "transcriptional groups (9313 genes)" were abundantly annotated among the 25 functional groups in the KOG database. Nuclear structure and cell motility accounted for the least annotated functional group, with 992 and 219 genes, respectively.

#### 3.3.3 Doum palm gene ontology (GO) functional classification and predicted CDS quality

**Fig 3:**
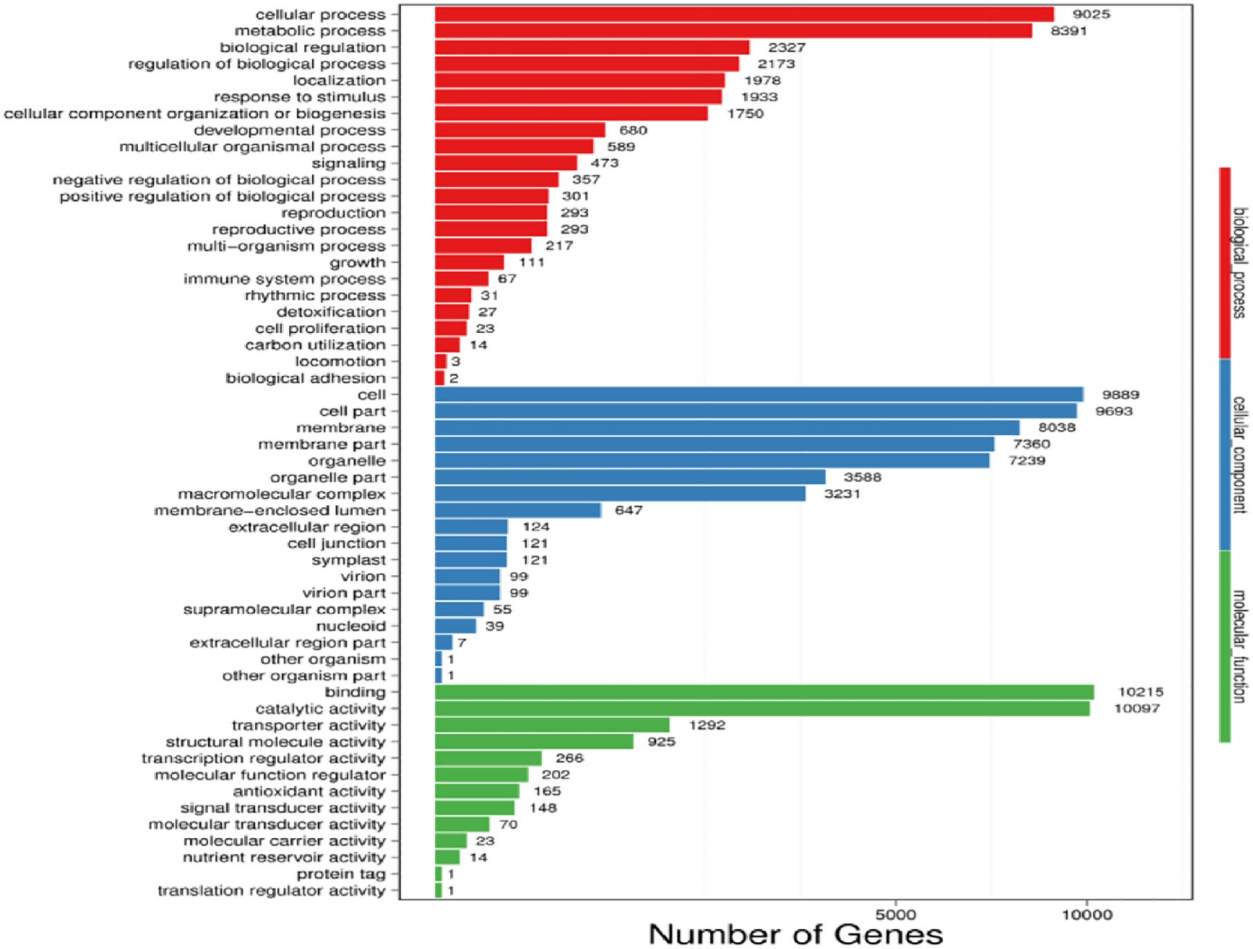
Functional classification of GO term. The abscissa and vertical coordinate depict the amount of Unigenes and GO term functional categories, respectively. Different color represents categories of GO term function.

Following these quality checks, the unigenes were annotated with biological roles. Regarding biological function, the bulk of the annotated unigenes was found to be linked with the cellular process (9025 genes), metabolic process (8391 genes), biological regulation (2327 genes), and regulation of biological processes (2173 genes). After cells and cell parts coded 9889 and 9693 genes, respectively, the membrane ranking third coded for 8038 genes while the membrane part ranked fourth coded for 7360 genes ranked among cellular components. Most unigenes exhibited binding (10,215 genes) when tested for molecular function and catalytic activity (10097 genes). Additionally, 1292 and 925 genes were involved in transporter and structural molecule activity, respectively. Fig 3 presents the GO term functional classification.

**Fig 4:**
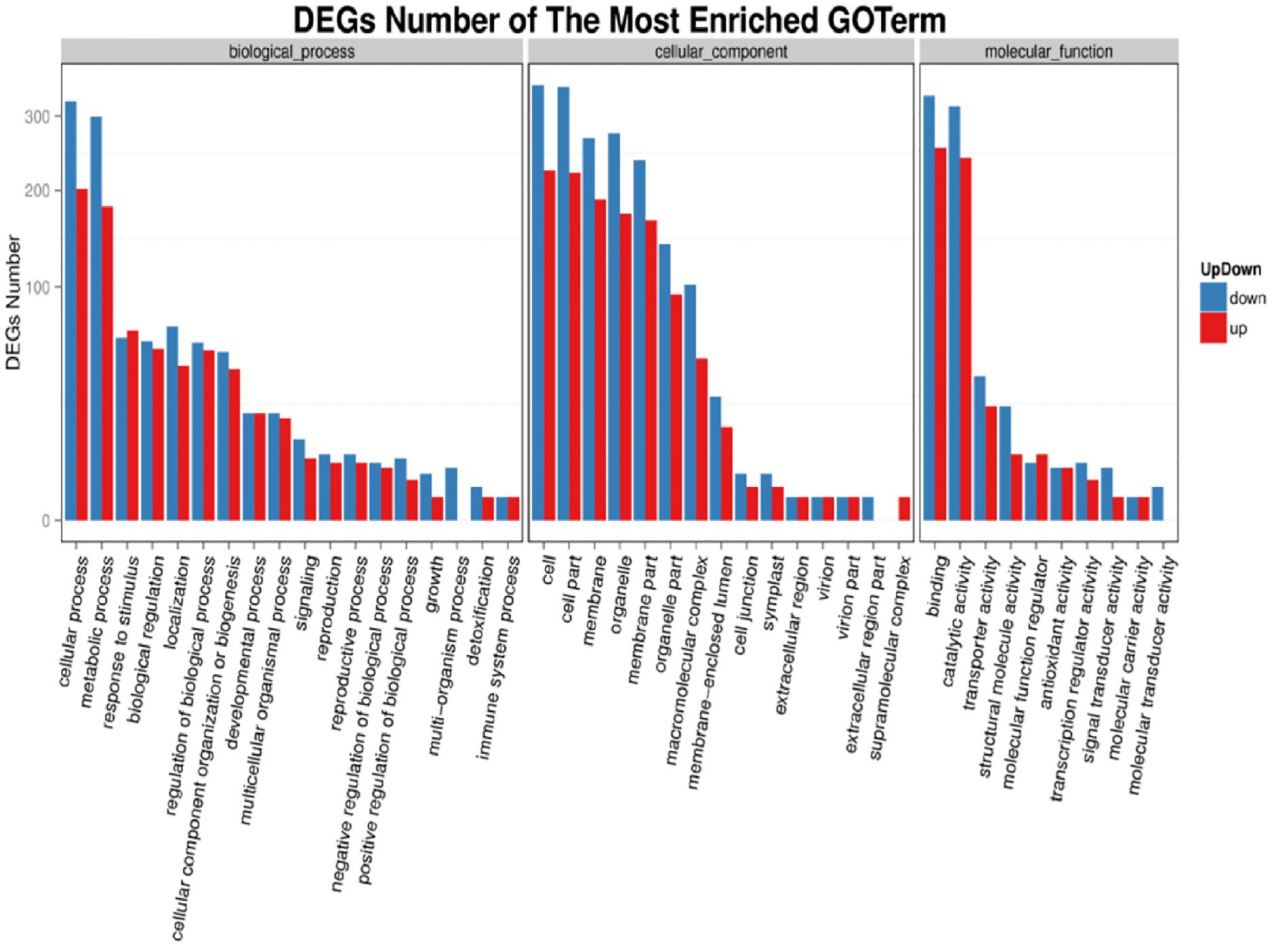
Doum palm GO terms categorized up-and down-regulated genes. The abscissa and vertical coordinate depict the GO term and how the genes were regulated, respectively.

Most DEGs were down-regulated, as evidenced by the enrichment of GO terms in the D control sample set against drought samples (Fig 4). The most down-regulated biological categories were binding and catalytic activity, cellular component and metabolic processes, and cells and their parts.

In the biological category, the most up-regulated expression were those connected to cells and their constituent parts; in the cellular component category, the utmost up-regulated processes were those linked to cells and their constituents; and in the cellular activity and cellular component categories, respectively, the most up-regulated processes were those related to catalytic activity and binding (Fig 4). The CDSs result of the unigene is presented in Table 5.

### 3.4 Classification of TFs in doum palm and identified SSR

#### 3.4.1 TFs in doum palm

The DEGs analysis of doum palms under well water and drought stress circumstances revealed the presence of fifty-eight (58) TFs families. The leading group of TFs was the MYB and MYB-related family, accounting for 683 and 502 genes, respectively, followed by FAR1 (378), C3H (315), bHLH (256), and WRKY (242), whereas other TFs families identified were those belonging to the AP2-EREBP (230), NAC (202), G2-like (176), and ARF (171) (S5 Fig). The MYB and MYB-related family accounted for the highest TF in doum palm, while HRT, GRF, and NOZZLE families were identified as the least. S5 Fig depict this study’s identified TFs in doum palms.

#### 3.4.2 Identified SSRs in doum palm

The popularity of SSR markers can be attributed to their ease, speed, low cost, and reproducibility. Based on the sequences of the functional genes of interest, SSR markers can be used in molecularly-assisted selection breeding, allowing for the investigation of genetic variation without identifying biases that arise in non-gene areas [30]. The majority of SSRs detected were dinucleotides (41.28%) and mononucleotides (34.1%), followed by trinucleotide and pentanucleotide repeats accounting for 20.64% and 1.79%, respectively (S6 Fig). The AG/CT repeat was the most prevalent SSR among mononucleotide repeats, followed by the AT/AT repeat. AGG/CCT and AAG/CTT accounted for the most common repeats in the dinucleotide type.

### 3.5 Gene expression and DEGs analysis

Using the FPKM method, we calculated the relative expression of protein-coding genes [31]. Also, the expression levels were used to rank the six samples into three groups: those with high, medium, and low levels (FPKM > = 10, FPKM 10, and FPKM 10). The control sample (M) (8515 genes) and stress sample (N) (8486 genes) had the most highly expressed genes, according to an analysis of the genes with FPKM > = 10 (S7 Fig).

**Fig 5:**
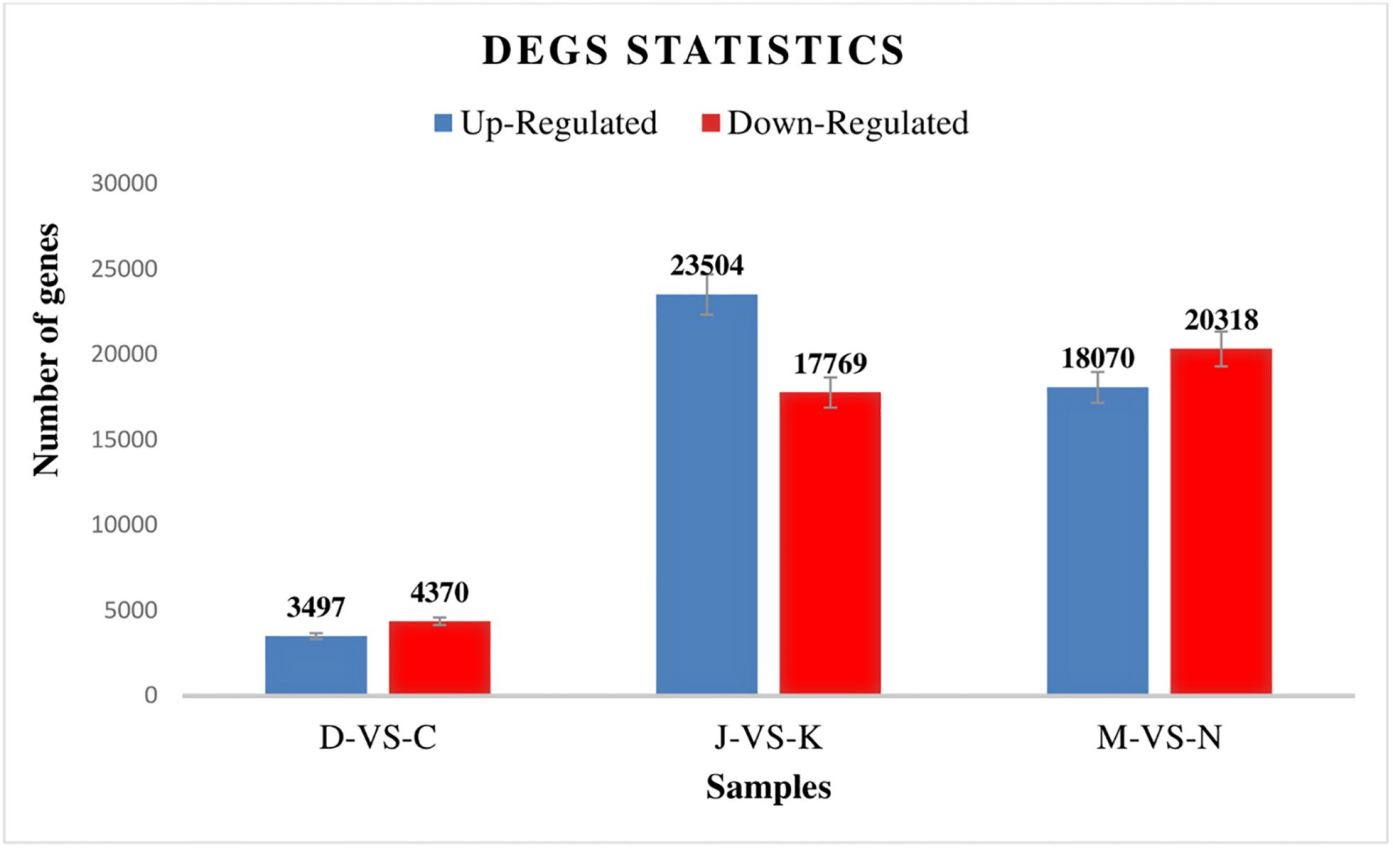
Genes differentially expressed in doum palm. NB: D, J, and M are well water samples; C, K, and N are stress-treated samples.

Differential expression of genes between well-watered and stressed-treated doum palms was analyzed to help us find novel candidate genes involved in water scarcity stress resistance. There were 87528 DEGs found between the control and tree treatment groups out of a total of 193,167 unigenes (Fig 5). Up-regulated DEGs accounted for 45071unigenes, and 42457 unigenes accounted for down-regulated DEGs.

### 3.6 qPCR results validating the RNA-Seq data

**Fig 6:**
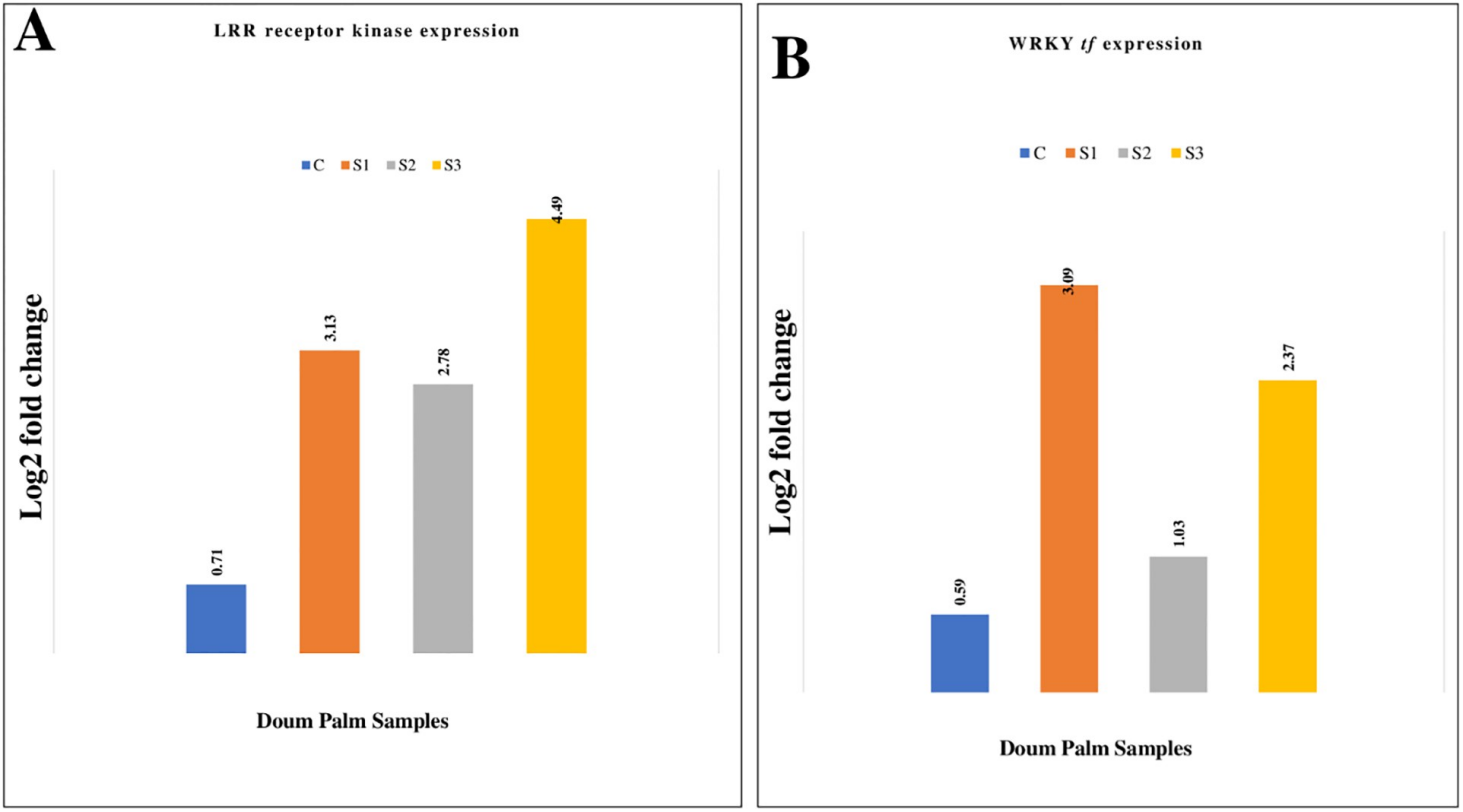
(A) LRR receptor-like gene and (B) WRKY tf gene expression in doum palm. Relative quantifications of leucine-rich repeats (LRR) receptor-like serine/threonine-protein kinase and WRKY transcription factor genes in 65 days stressed doum palm samples (Fold change in log 2 ratio). NB: C = control sample, S1 = stressed sample1, S2 = stressed sample 2, and S3 = stressed sample 3.

**Fig 7:**
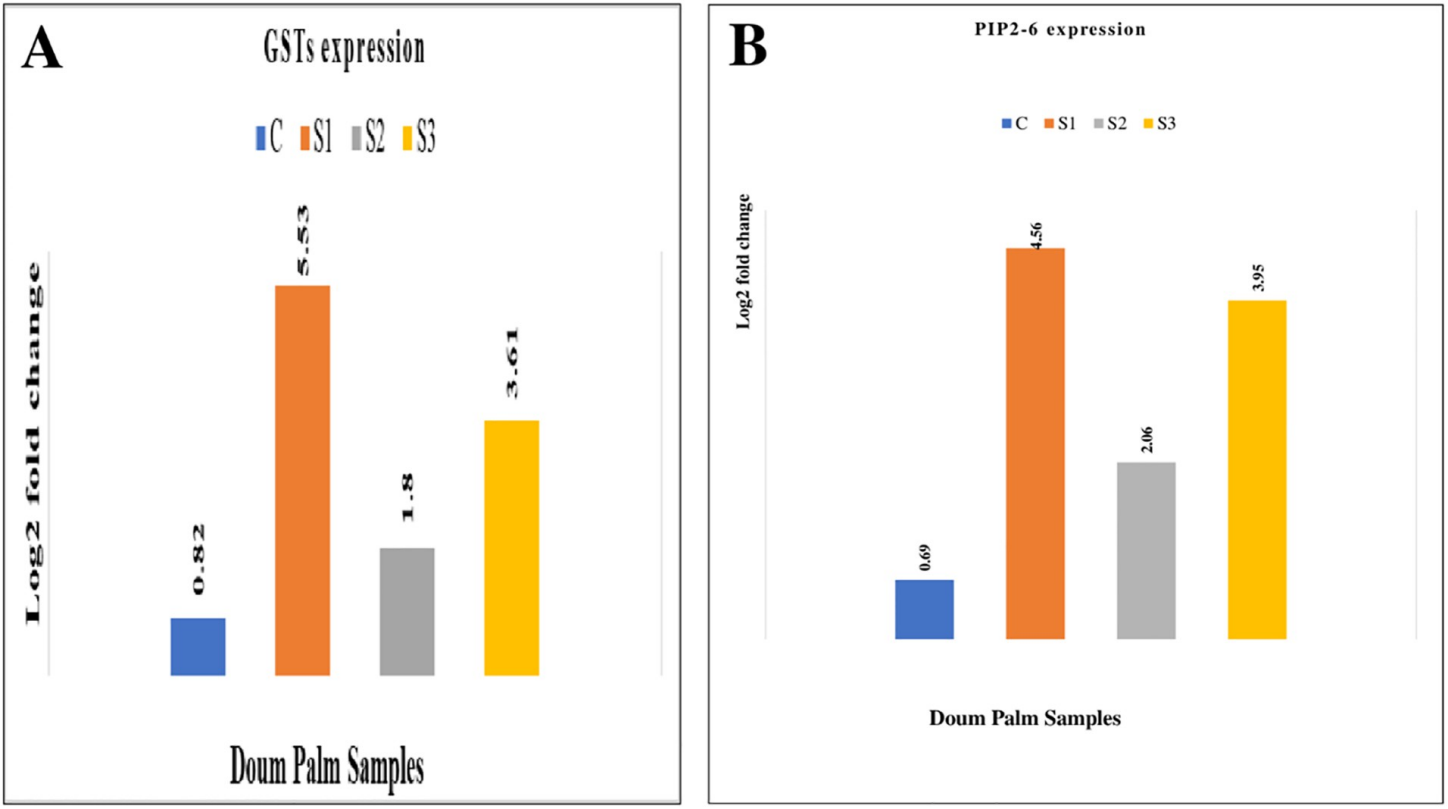
(A) GSTs gene and (B) PIP2-6 gene expression in doum palm. Relative quantifications of glutathione S-transferases T1 (GSTs) and aquaporins PIP2-6 genes in 65 days stressed doum palm samples (Fold change in log 2 ratio). NB: C = control sample, S1 = stressed sample1, S2 = stressed sample 2, and S3 = stressed sample 3.

Four DEGs were arbitrarily chosen for relative quantitative analysis to validate the RNA sequencing data. These selected DEGs exhibited considerable up-regulation of all selected DEGs in the RNA-Seq study. Drought-treated plants exhibited strong upregulation of the four unigenes after the qPCR assay. Figs 6A, 6B, 7A, and 7B display that qPCR and RNA sequencing data showed consistent patterns (up-regulation) of unigene expression.

## 4 Discussion

### 4.1 Perspectives on the doum palm de novo transcriptome assembly and annotation

Several genes in a plant regulate and modulate the complicated biological process known as drought resistance. The cellular response processes to water deficit stress were investigated in the doum palm, a plant without a reference genome, utilizing an all-encompassing approach. Genomic and transcriptomic data for doum palms are still missing to provide a complete picture of the plant’s genetic history or functional genes. As a result, there are many unannotated unigenes; in the current analysis, just 66.63 percent of all unigenes had annotations in any database. Annotation results from filtered reads used to sequence reported species’ transcriptomes revealed that 83.48% of unigenes were present in public databases. This includes transcripts from common vetch [32]. Compared to data on common buckwheat, which had 39.33% of unigenes annotated in the GO catalog, the fraction of annotated genes in the doum palm genome is significantly more significant [33]; likewise, the *Taxus* species had 8426 and 12,642 annotated unigenes in the KEGG and GO term databases, respectively [32]. These findings suggest that a substantial amount of transcriptome data still needs further studies for organisms that do not contain a reference genome, such as the doum palm. The unmatched unigenes probably represent doum palm-specific genes. Transcriptome outcomes from this work were similar to those from other previous transcriptomic investigations of crop species, including maize [34], mulberry (*Morus* L.), [35], and *Ammopiptanthus mongolicus* [36].

The species with the best hits in the annotated sequences were the date palm (*Phoenix dactylifera*), the oil palm (*Elaeis guineensis)*, and the *ananas comosus*. Doum palm is the nearest species to date, and oil palm with sequenced genomes belongs to the *Arecaceae* family. Therefore, these findings are consistent. The genomic sequences for date palms and oil palms have been publicly available since 2013 [37, 38]. It is anticipated that when the doum palm genome, a member of the family *Arecaceae*, is accessible, a comparison analysis would further illuminate the differences in their features and the evolutionary links.

### 4.2 KOG functional classification analysis

The most significant fraction of genes in the KOG analysis were classified as having "general function prediction," "signal transduction mechanisms," "posttranslational modification," "protein turnover," and "chaperones" categories. These data support the findings of [39], who tackled the difficulties associated with *de novo* sequence analysis of the sweet potato genome. Stress from drought triggers a cascade of molecular activities in plants, including signal transduction, the cellular process that responds to a physical or chemical stimulus [40, 41]. When a trigger is detected, the plant responds by changing the enzyme activity of proteins, striking an equilibrium between protein production and breakdown [42]. Chaperone proteins, which facilitate protein folding as a chemical process due to extreme environmental stress, also help prevent or repair damage caused by misfolded proteins [43]. Recent research has divided drought-inducible genes into two groups: functional proteins and regulatory proteins. Proteins such as those found in the late embryonic abundant (LEA) fraction, chaperones, dehydrin, detoxifying enzymes, and osmolytes mediate abiotic stress tolerance; on the other hand, a network of regulatory proteins controls genes involved in drought resistance [43].

### 4.3 GO term functional category reveal in doum palm under stress

In agreement with several prior studies [44], transcripts associated with "binding" and "catalytic activity" were over-represented under the Molecular function category of Gene Ontology, as revealed in the current study. Gene interaction is a common and crucial phenomenon that controls many biological processes. In response to drought stress, plants may express downstream target genes better or accumulate microRNAs if specific genes are activated. Therefore, gene binding activity regulates plant stress responses [45].

Generally, there was more than one unigene present in a given category. The two main categories in the cellular component group were "Cell" and "Cell part." This finding conforms to that of [46], in which Cell and Cell components accounted for the most significant functional category of unigenes annotated in the GO database. Similar GO terms in retort to external environmental factors have also been observed in chickpea transcriptome investigations [45, 47, 48].

### 4.4 Doum palm KEGG pathway analysis for DEGs

KEGG enrichment analyses on DEGs revealed that several differentially expressed genes were most strongly enriched, including cellular activities, environmental information processing, genetic information processing, metabolism, and organismal systems, indicating various functional activities/processes in leaves of doum palm under stress. These annotations are an excellent resource for learning about plants, mainly when cultivating climate-smart crops [35]. The regulation of multiple critical metabolic processes is intrinsically linked to the KEGG pathways enrichment analysis of doum palm, as similar findings were observed in rice [49, 50]. Our results also corroborated the findings of prior studies showing that environmental adaption was enhanced after water stress treatment [51, 52].

### 4.5 Drought-induced increases TFs activities in doum palm

In response to stressful conditions, plants use transcription regulation to temporarily and spatially regulate the expression of their target transcription genes [53, 54]. According to recent research [55], TFs significantly contribute to plant drought resistance by controlling the transcription of downstream genes to increase plant stress tolerance. This investigation will help researchers pinpoint and categorize TFs, providing a deeper insight into the molecular processes behind doum palms’ drought tolerance. The leading TF families of doum palm, which include the myeloblastosis (MYB) and MYB-related, WRKY, NAC family (NAM, ATAF, and CUC), FAR-RED-IMPAIRED RESPONSE1 (FAR1), B3, and bZIP, were represented by DEGs encoding TFs. Many of these identified TFs families have been linked to improved plant stress resistance [56]. Accurate modulation of gene activities by TFs is essential for many plant functions, such as responding to environmental stresses [57, 58]. Multiple transcription factor genes, including ethylene-responsive element binding protein, bZIP, the myelocytomatosis tfs, NAC, and WRKY, have been associated with plant reactions to stress [59].

The current investigations are comparable to those of [60], who conducted a DEGs analysis on drought-stressed Giant Juncao and discovered that 56 TFs families were represented. In retort to a drought signal, the plant initiates a series of signaling cascades that ultimately leads to the activation of a TF. Furthermore, in response to a water deficit, an active TF attaches to a cis-acting site in the interested gene, which then triggers transcription of a specific response gene further down the gene’s regulatory pathway [56]. This research uncovered various transcription factors with varying expression levels crucial for withstanding drought stress.

The MYB-related class of TF is among the numerous plant TFs and is essential for coping with various adversities such as drought, light signaling, and stress. Increased activity of MYB family genes in transgenic rice, as seen with OsMYB6, improves the plant’s resilience to water stress [61]. According to the research of [62], the MYB and MYB-related families can regulate drought tolerance in plants.

Numerous studies have linked WRKY TF to drought tolerance [63]. At least 17 rice WRKY genes were triggered by drought using massively parallel signature sequencing (MPSS) technology [64]. For example, [65] discovered that transgenic tobacco plants expressing TaWRKY44, a member of the WRKY TFs, were extra tolerant to water deficit and salt than non-transgenic tobacco crops. These outcomes indicate that TaWRKY44 may have a beneficial regulatory function in retorts to drought, salt, and osmotic conditions by expediting the effective removal of ROS by promoting the production of genes involved in dealing with stress. Similarly, the expression of CsWRKY2, like that of other WRKY TFs, was shown to be up-regulated in response to exogenous ABA, indicating that this gene contributes to tea plants’ ability to withstand water stress [66].

Identification and investigation of TF types in plants and other organisms benefit significantly from de novo analysis. Most bHLH family members have been linked with water deficit conditions [67]. For instance, the rice, maize, and wheat genomes have 183, 23,1, and 571 members of the bHLH family, respectively [54], while *Arabidopsis* has 162, Chinese cabbage has 230, *Brachypodium distachyon* has 146, and apple has 175 [67, 68]. And therefore, breeding crops to thrive in water-poor environments would benefit significantly from a better understanding of which TFs are involved in this process and which genes they regulate.

### 4.6 Generic SSR makers in doum palm under stress

Research on the transcriptome can reveal genetic markers, making them an excellent choice for molecular cultivation. Due to their rapid amplification levy and good transferability between organisms, markers derived from the transcriptome have proven superior to those derived from non-transcribed areas [69]. Although single-nucleotide polymorphism (SNP) markers are best for understanding trait architecture [70], simple sequence repeats (SSRs) markers and other genes/length polymorphism-based markers are preferable for breeding applications. Comparable to what was described in previous plant research [71, 72], the preponderance of SSRs identified in doum palms were dinucleotides (41.28%), followed by mononucleotides (34.1%). Our findings are consistent with [73] *de novo* transcriptome assembly and gene quantification investigation in several moth bean tissues (*Vigna aconitifolia* Jacq.). Previous research has also reported on the function of SSRs in counterpoise to various abiotic conditions [74, 75].

### 4.7 Doum palm gene expression via transcriptomic profiling

Due to variations in the number of expressed genes and the level of gene activities in samples, the sample expression value of FPKM can be divided into various intervals. Statistics on gene expression levels are calculated and presented as stacked histograms of data from multiple time points. When [76] analyzed the groundnut transcriptome globally, they found that transcription factors, biological activities, and related pathways were similarly distributed across the genome, in line with our findings.

#### 4.7.1 Analysis of selected doum palm transcripts based on their response to drought

The selected transcripts in the qPCR analysis include leucine-rich repeats (LRR) receptor-like serine/threonine-protein kinase, WRKY transcription factor, glutathione S-transferases T1 (GSTs), and aquaporins PIP2-6, were found to be significantly up-regulated in doum palm based on the expression analysis data. These genes/gene products or regulatory sequences have been linked to improved drought tolerance using a combination of in-depth literature reviews and extensive searches of publicly available databases.

LRR-RLKs are involved in numerous processes vital to plant life, including growth and development and detecting signals relevant to retorts to abiotic conditions [40]. For example, LRR-RLKs are crucial in controlling how plants react to water deficit and salinity conditions [77, 78]. In another example, drought, osmotic stress, auxin, and abscisic acid stimulated the Arabidopsis LRR-II type RLK genes. These genes affect the crop’s response to external environmental influences [79].

The expression of rice LRR-RLKs (OsSIK1, OsGIRL1, OsLP2) was regulated in response to a wide range of stresses, including salinity, water deficit, abscisic acid, salicylic acid, and hydrogen peroxide, suggesting that these genes may function in various signaling networks that drive development and stress responses [15, 80]. Multiple studies have revealed that LRR-RLKs respond to drought by increasing their expression levels. In response to water deficit and salt treatment, for example, the expression of PnLRR-RLK27 in the Antarctic moss Pohlia nutans significantly up-regulated transcript levels of essential transcription factors and stress-related genes [81]. The Pkinases GhMKK1 and GhMKK3 have also been linked to cotton’s resilience to salinity and water deficit [82].

The WRKY protein family is among plants’ most numerous transcription factors (TFs). A highly conserved WRKY domain made up of the DNA-binding heptapeptide WRKYGQK, and the zinc finger binding motif spanning roughly 60 amino acids in length is a defining feature of WRKY TF [83]. WRKY is a member of the transcription factor superfamily and is involved in numerous biological activity processes during the plant’s development and growth. Still, it is crucial in the defense reaction to harsh environmental conditions by modulating the transcription of genes involved in responding to those stresses through phytohormones. Moreover, extensive research shows that WRKY enhances the plant’s retort to water shortage conditions [84–86].

The expression of WRKY TFs varies in response to environmental factors like salinity, water deficit, pathogen inoculation, the application of growth hormones, and oxidative stress, according to several scientific investigations utilizing transcriptome analysis [63]. Consistent with our findings, [87] demonstrated increased expression of 15 EgWRKYs TFs gene candidates in oil palms after drought, salinity, and heat exposure. Water-stress-induced upregulation of numerous transcription factors (TFs) may result in higher expression of genes of interest. [88] demonstrated that co-expression of AtWRKY28, a member of the WRKY TFs family, boosted the activities of numerous genes of interest in *Arabidopsis* in response to abiotic conditions such as drought, salt, and oxidative stress. To a large extent, WRKY TFs appear to regulate water deficit conditions via altering the osmotic equilibrium, ROS sifting, and the expression of several drought-related genes [89]. WRKY TF’s potential led to its selection as one of the candidate genes for sequencing data.

Plants utilize many genes in water status maintenance in response to various environmental stresses. Many different cellular processes in plants also depend on glutathione S-transferases (GSTs), an enzyme family with many different roles, including shielding cells from oxidative stress damage and scavenging ROS due to water deficit duress [90–92]. ThGSTZ1 transient increased expression in transgenic T. hispida, for instance, elevated GST and GPX activities and boosted ROS scavenging abilities in response to NaCl and mannitol treatments [91]. These findings, consistent with the current study’s expression pattern, indicate that GSTs can increase plant resistance to drought and salinity by boosting ROS scavenging.

Many plant species have undergone genome-wide functional characterization of the glutathione S-transferases (GSTs) gene family. A recent study published by [93] found that cold, heat, drought, salt, and osmotic stress led to significant upregulation in pepper GST transcript expression and overall pepper GST activity, further supporting the current finding. As a result of its demonstrated association with several pathways involved in the control of plant stress, doum palm RNA-Seq data validation with GST transcripts was of significant interest.

As a subfamily of the MIP superfamily, aquaporins (AQPs) create channels in membranes that are permeable to water and other tiny neutral substances. It has been established that abiotic stress causes plants to up-regulate AQPs [94–96]. The functional characterization of AQPs in many crop species revealed that this gene had improved resistance to abiotic stress [97, 98]. It is well established that the AQP family, of which the PIP proteins are a subset, plays crucial roles in water transport across the plasma membranes of many different crop species [99, 100].

In line with the findings of this study, the PIP2-6 genes showed significant expression under increased water duress in a prior investigation [101]. Similar results were found in earlier publications on chickpeas [102], which examined variations in water transport networks in the root apoplast and found that PIP2-6 gene expression was similarly significantly elevated. Considering the possible role of the aquaporin (AQP) genes family in harsh environmental condition resistance, specifically in its active involvement in water transport and desiccation resistance, the aquaporins PIP2-6 were chosen for use in confirming the RNA-Seq data.

## 5 Conclusion

Here, we present the first transcriptome data from doum palms that allowed us to characterize the genes involved in their response to drought. *De novo* assembly yielded 93,907 unigenes, and seven functional databases provided annotations for 57,941 genes. Deciphering the response mechanism of doum palms under drought stress can be aided by studying the expression of DEGs and finding transcription factors/genes related to drought. This study sheds light on the genetics of drought tolerance adaptation in doum palms and identifies prospective genetic resources for abiotic stress resistance in plant development. From a drought tolerance perspective, this study shows that doum palm resources can be exploited as a breeding crop that can thrive well during drought stress. This study has made the first comprehensive molecular/transcriptome resources available to the scientific community to facilitate increasing studies on doum palm species.

## Supporting information

S1 Fig NR annotation indicating the range of species. (DOCX)

S2 Fig Total length of doum palm assembled unigene. (DOCX)

S3 Fig Annotation matrices for NR, KOG, KEGG, Swissprot, and Interpro. (DOCX)

S4 Fig The range of doum palm CDS lengths. (DOCX)

S5 Fig Doum palm transcription factor (TF) family classification. (DOCX)

S6 Fig Detected SSRs in doum palm. (DOCX)

S7 Fig Gene expression distribution per sample. (DOCX)
