# Supplementary material for: De novo transcriptomic analysis of Doum Palm (Hyphaene compressa) revealed an insight into its potential drought tolerance
Source: PLoS One. 2024 Mar 12;19(3):e0292543. doi: 10.1371/journal.pone.0292543 (PMC10931477; doi:10.1371/journal.pone.0292543)

**DE NOVO TRANSCRIPTOMIC ANALYSIS OF DOUM PALM (HYPHAENE COMPRESSA) REVEALED AN INSIGHT INTO ITS POTENTIAL DROUGHT TOLERANCE**


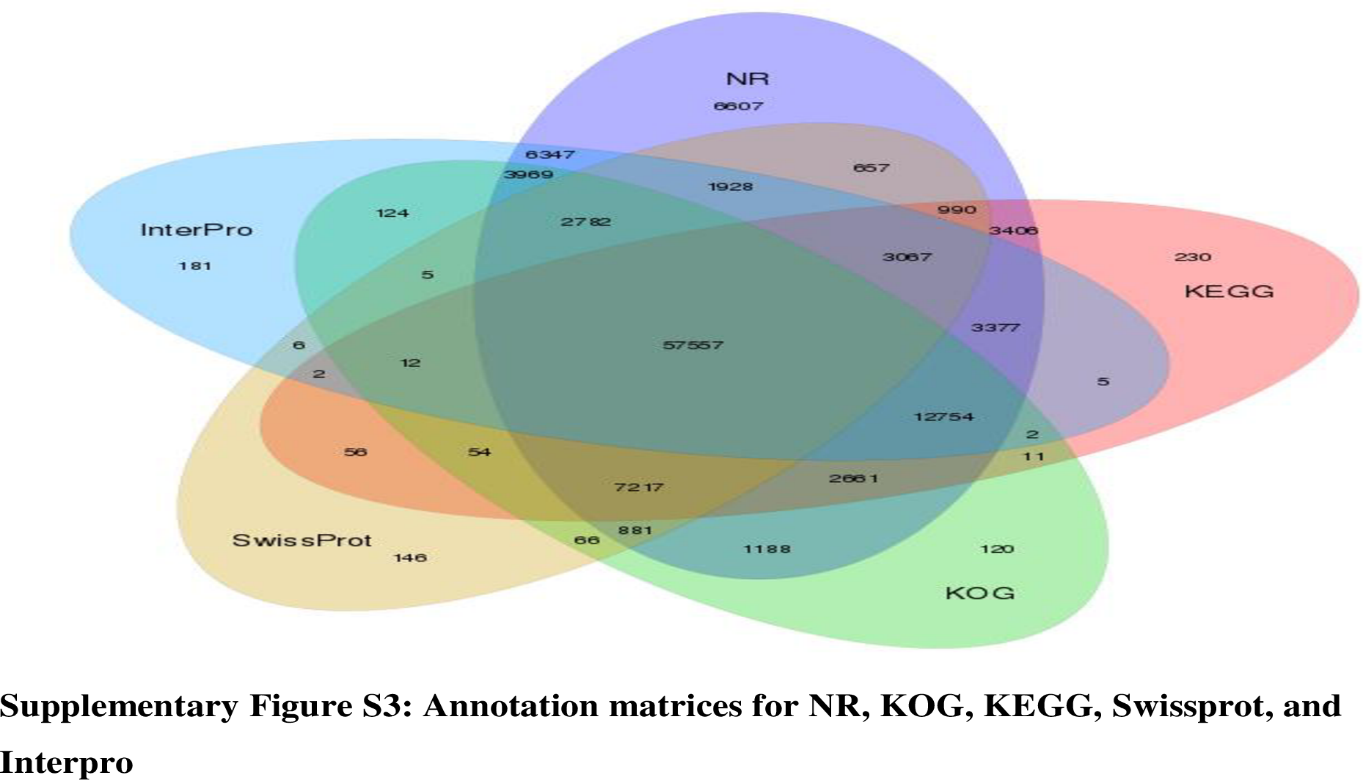

Supplement: S3 Fig — (DOCX) [file pone.0292543.s003.docx]
